# Targeting Endoplasmic Reticulum Stress as an Effective Treatment for Alcoholic Pancreatitis

**DOI:** 10.3390/biomedicines10010108

**Published:** 2022-01-05

**Authors:** Hui Li, Wen Wen, Jia Luo

**Affiliations:** 1 Department of Pathology, Carver College of Medicine, University of Iowa, Iowa City, IA 52242, USA; hui-li@uiowa.edu (H.L.); wen-wen@uiowa.edu (W.W.); 2 Iowa City VA Health Care System, Iowa City, IA 52246, USA

**Keywords:** alcohol abuse, cell signaling, FDA-approved drugs, oxidative stress, therapy

## Abstract

Pancreatitis and alcoholic pancreatitis are serious health concerns with an urgent need for effective treatment strategies. Alcohol is a known etiological factor for pancreatitis, including acute pancreatitis (AP) and chronic pancreatitis (CP). Excessive alcohol consumption induces many pathological stress responses; of particular note is endoplasmic reticulum (ER) stress and adaptive unfolded protein response (UPR). ER stress results from the accumulation of unfolded/misfolded protein in the ER and is implicated in the pathogenesis of alcoholic pancreatitis. Here, we summarize the possible mechanisms by which ER stress contributes to alcoholic pancreatitis. We also discuss potential approaches targeting ER stress and UPR in developing *novel* therapeutic strategies for the disease.

## 1. Acute and Chronic Pancreatitis

Pancreatitis is a common inflammatory disorder of the pancreas, associated with high mortality and healthcare burdens worldwide [1,2]. It mainly consists of two forms: acute pancreatitis (AP) and chronic pancreatitis (CP). AP is the most frequent cause of gastrointestinal disorders requiring hospitalization in the US, and its associated inpatient care cost is approximately USD 2.6 billion annually [2,3,4]. Although less frequent, CP also causes significant morbidity and financial burden [3]. Additionally, the incidence of pancreatitis differs with age and gender. The risk of developing AP increases with age [5,6], whereas CP is more common in middle-aged individuals [2]. Furthermore, AP does not appear to differ between men and women [6]; however, CP is more common in men than in women [2,7]. AP and CP share a significant portion of clinical manifestations and phenotypes, but also have distinct morphological and imaging features.

AP is characterized by sudden abdominal pain, elevated levels of pancreatic enzymes in the blood, and pancreatic inflammation [8,9]. Depending on the clinical features, AP can be classified into mild, moderate, or severe forms. The most common form of AP is mild AP, which can be self-treated within weeks. However, the moderate and severe forms can progress into necrotizing pancreatitis, which has a 20–40% mortality rate [10]. A variety of long-term sequelae have been reported that can persist beyond hospital admission for AP. AP may increase the risk of other pancreatic disorders, including CP, exocrine pancreatic insufficiency (EPI), pancreatic cancer (PC) and diabetes mellitus (DM). In total, 17% of AP patients are re-admitted after their first episode for recurrent pancreatitis with about 8% of patients developing CP [11,12]. Approximately one quarter to one third of AP patients develop EPI during the follow-up period [13,14]. The prevalence of EPI following AP is higher with the severe form than with the mild form, and it is higher in patients with an etiology of alcohol than one of gallstones [14]. AP patients often develop prediabetes and/or DM after being discharged from the hospital [15,16]. The diagnosis of AP increases the risk of PC, which in turn increases the number of recurrent episodes of AP [17,18].

CP is believed to result from the recurrence of AP, leading to chronic pain, pancreatic atrophy, duct strictures and calcifications [19,20]. Although less common than AP, CP significantly affects patients’ quality of life due to irreversible, debilitating injury to the function of the pancreas. CP is also associated with other pancreatic diseases. It has been reported that CP increases the risk of EPI [21,22], PC [23,24] and DM [25,26]. The high disease burden of AP and CP emphasizes the importance of identifying predisposing factors, understanding pathogenesis, and developing therapeutic intervention for these diseases.

## 2. Alcohol Consumption and Pancreatitis

Alcohol exposure is a known etiological factor for both AP and CP. Epidemiological studies have shown that excessive alcohol consumption is the second leading cause of AP after gallstones [1,27] and is the most prevalent risk factor for CP [28]. Alcohol abuse is also a risk factor for the recurrence of AP and increases the chance of the progression of AP into CP [11,29]. Although alcohol can contribute to the initiation and progression of pancreatitis, only a small number of heavy alcohol drinkers develop the disease, suggesting that other disposing factors are involved in the development of alcohol-related pancreatitis [7,30,31,32].

The association between alcohol consumption and pancreatitis is evaluated predominantly by self-reported survey studies. Corrao et al. conducted a meta-analysis of studies published from 1966 to 1995 and showed that the risk of pancreatitis monotonically increased with increasing alcohol consumption [33]. Consistent with this finding, Irving et al. analyzed research published from 1980 to 2008 and confirmed a monotonic dose–response relationship between alcohol consumption and the risk of pancreatitis, with a threshold of four drinks daily that significantly increased the risk of pancreatitis [34]. Similarly, more recent studies indicated that prolonged use of alcohol with a threshold level of 4–5 drinks per day was required for an increased risk of pancreatitis [19,31,34,35,36]. In addition, the amount of recently consumed alcohol was shown to determine the severity of the first episode of acute alcoholic pancreatitis [37]. In the absence of long-term use, binge drinking alone did not increase the incidence of AP [38]. Regular consumption of alcohol at lower levels, however, appeared to have an inconsistent effect on pancreatitis. Some reported that low levels of alcohol drinking (<50 g per day) increased the recurrence of AP and accelerated the progression of CP [39,40]. Others found that mild or moderate drinking was inversely associated with an increased risk of pancreatitis [41].

In contrast to prolonged heavy alcohol consumption, which has been known as a risk factor for pancreatitis, alcohol abstinence has been shown to slow down the progression of pancreatitis and reduce the recurrence of AP. For example, withholding from drinking resolved abdominal pain and slowed the deterioration of pancreatic function in chronic heavy drinkers [42]. Abstinence after the first episode of AP minimized the number of recurrent attacks [43]. Similarly, in an effort to determine the risk factors associated with recurrent pancreatitis, Pelli et al. (2008) showed that abstinence from alcohol protected against the recurrence of AP [44].

Alcohol can also act as a co-factor to increase the sensitivity of the pancreas to the detrimental effect of other risk factors, including environmental and dietary factors [45]. Cigarette smoking is an independent risk factor for a number of pancreatic disorders, including AP [46], CP [47] and PC [48,49]. Alcohol drinking can accelerate the progression of cigarette-smoking-related pancreatitis and vice versa, suggesting a synergistic interaction between alcohol and smoking in the development of the disease [36,50,51,52]. Hypertriglyceridemia, referring to an elevated blood level of triglycerides often resulting from high dietary fats, is another important cause for pancreatitis [53,54,55] and is present in many alcoholics [56,57]. Excessive alcohol consumption has been suggested to be associated with hypertriglyceridemia-induced pancreatitis [58,59].

The risk of alcoholic pancreatitis can also be altered by genetic modifiers. The *CLDN2* (Clauding 2) gene encodes a tight junction protein-regulating cation and water transport of epithelial cells. It is normally expressed in pancreatic duct cells but not acinar cells [60,61]. In a genome-wide study, a *CLDN2* risk allele, which is associated with an abnormal expression of CLDN2 protein in pancreatic acinar cells, was identified as a risk factor that interacted with alcohol consumption to accelerate the progression of chronic pancreatitis [62]. In another genome-wide association study, an inversion of the *CTRB1–CTRB2* (chymotrypsin B1 and B2) locus led to both the imbalanced expression of CTRB1 and CTRB2 and an increased risk for both alcoholic CP and non-alcoholic CP [63].

Racial/ethnic differences are another susceptibility factor that can alter the risk of alcoholic pancreatitis. A population study using nationwide inpatient samples from the racially diverse US population between 1988 and 2004 demonstrated that Black people had the highest frequency of alcohol-related pancreatitis [64]. Another study using data collected by the North American Pancreatitis Study Group from 2000 to 2014 found that Black people were more likely to be diagnosed with CP than White people, likely because of alcohol consumption and smoking being more frequent in Black people [65]. In a number of studies conducted in the Asian population, a dose–response relationship between alcohol and pancreatitis was revealed [66,67,68]. The impact of ethnicity on the risk of alcoholic pancreatitis in these Asian studies was suggested to be related to the genetic polymorphism of alcohol metabolism enzymes. Genetic variant alleles of the aldehyde dehydrogenase-2 gene (ALDH2*2) and alcohol dehydrogenase-1B gene (ADH1B*2), which are associated with the accumulation of toxic acetaldehyde after alcohol drinking, were predominantly found in East Asians [69,70,71].

## 3. Animal and Cell Culture Models for Alcoholic Pancreatitis

Epidemiologic studies have indicated that alcohol can act as a mild initiator or a robust modifier that sensitizes the pancreas to the insult of other risk factors during the development of pancreatitis. To understand the mechanisms underlying the pathogenesis of alcohol-related pancreatitis, many animal and cell culture models have been established. These experimental models have recapitulated the clinical features of alcohol-related pancreatitis, facilitated our understanding of the pathology, and provided opportunities to test potential therapeutic treatments for the disease.

Consistent with epidemiologic studies, alcohol alone, either by acute exposure (77) or by chronic feeding [72,73,74], is not sufficient in inducing pancreatitis-like features in rodent models. Recent studies have combined chronic exposure with binge drinking and found that alcohol, when acting as both the initiation and susceptibility factor, can cause pancreatic injury, mimicking pancreatitis. Binge alcohol exposure by intragastric intubation for 10 consecutive days (5 g/kg/day, 25% ethanol *w*/*v*) caused pancreatic edema, acinar cell death and moderate fibrosis in C57BL mice [75]. Mice receiving a liquid alcohol diet for two weeks followed by binge alcohol exposure by oral gavage for 3 days (5 g/kg/day, 25% ethanol *w*/*v*) displayed more severe injury and inflammation in the pancreas [76]. A 10-day feeding of a liquid alcohol diet plus a single binge ethanol exposure was found to lead to pancreatic edema and inflammation in C57Bl/6 mice [77,78]. The chronic plus binge model may be of clinical relevance due to the similarity of the drinking pattern to that of many alcoholic patients who have a history of chronic alcohol consumption and tend towards heavy episodic drinking [79,80,81]. In fact, the chronic plus binge exposure has also been used in animal models for alcoholic liver disease (ALD), as it causes significantly higher elevation of serum alanine aminotransferase (ALT) and aspartate aminotransferase (AST) levels and hepatic histological features, compared with chronic alcohol feeding or binge exposure alone [77,82,83].

The detrimental effects of alcohol on the pancreas can result from the direct actions of toxic metabolites, acetaldehyde and fatty acid ethyl esters (FAEEs), via the oxidative and non-oxidative pathways, respectively. The oxidative metabolism of ethanol mainly occurs in the liver [84,85,86] and the level of acetaldehyde in the circulation is typically low [87,88], meaning organ damage in the pancreas by acetaldehyde is considered insignificant. In contrast, non-oxidative metabolism of ethanol by esterification with fatty acids, resulting in the formation of FAEEs, has been implicated in alcohol-induced damage to the pancreas. An autopsy study showed that the level of FAEEs and the activity of FAEEs synthase (enzymes responsible for the synthesis of FAEEs) are highest in the pancreas among all ethanol-damaged organs in acutely intoxicated individuals [89]. In fact, intra-arteria infusion of FAEEs in rats at concentrations comparable to those in human plasma only caused AP-like injury in the pancreas but not in other organs that are known to be susceptible to ethanol-induced damage, implying a role of FAEEs as a mediator in ethanol-induced pancreas-specific toxicity [90]. In a ethanol-induced AP rat model, the inhibition of oxidative ethanol metabolism increased FAEEs concentration in the plasma and pancreas and exacerbated pancreatitis-like injury, suggesting FAEEs are responsible for pancreatic damage in alcohol-related AP [91]. With in vitro and in vivo models for AP induced by low ethanol and fat, Huang et al. (2014) showed that 3-benzyl-6-chloro-2-pyrone (3-BCP), an inhibitor of carboxylester lipase (a FAEE synthase produced by pancreatic acinar cells), reduced FAEEs formation and alleviated exocrine pancreatic damage, demonstrating a crucial role of FAEEs in alcohol-related AP [92].

Alcohol can also act as a co-factor to sensitize the pancreas to the adverse effects of other susceptibility factors in the progression of pancreatitis. One physiologically relevant animal model for alcohol-related pancreatitis is the co-exposure of cholecystokinin (CCK) analogs and alcohol. CCK, an intestine hormone, is one of the most commonly used models to induce mild AP in rats [93,94,95,96] and a more severe form in mice [97,98,99,100], with a dose that is at least 10 times higher than physiological conditions. CCK analog-induced AP can recapitulate the pathologic features of human AP caused by scorpion venom and cholinergic toxins [101,102,103,104]. The co-treatment of alcohol can either reduce the threshold concentration of CCK analogs required to elicit a pancreatitis response or intensify the pathologic response of the pancreas. Pandol et al. (1999) demonstrated that alcohol exposure sensitized rats to pancreatitis induced by CCK-8 at physiological concentration, which by itself did not cause pancreatitis [95]. Quon et al. (1992) showed that chronic feeding with an alcohol diet exacerbated CCK analog caerulein-induced pancreatitis in rats, signified by greater increases in serum lipase level, interstitial edema and acinar vacuolization compared with animals treated with caerulein alone [105]. Repeated use of caerulein over time induced pathological features of the pancreas in rodents that mimicked human CP [106,107,108]. Alcohol exposure accelerated the progression of caerulein-induced CP in rats [108] and mice [109].

Another clinically relevant animal model is lipopolysaccharides (LPS)-induced alcoholic pancreatitis in rodents [110]. LPS are endotoxins derived from Gram-negative bacteria in the gut, which can be released to the blood to cause LPS-associated toxicity [111]. There have been reports of higher plasma levels of LPS in alcoholics [112,113] and an association between plasma endotoxin concentrations and the severity of human AP [114]. In rat models, LPS and alcohol exposure have been shown to cause a more severe pancreatic injury than LPS alone [110,115]. Withdrawal of alcohol after manifestation of LPS-induced pancreatitis in rats resulted in the resolution of pancreatic lesions, including fibrosis and cell death, whereas continued alcohol administration aggravated the injury [116]. In a rat model of alcoholic AP, alcohol increased the expression of LPS-induced proinflammatory factors in acinar cells, including TNFα, IL-6, IL-10 and IL-18 [117]. The elevated expression of these inflammatory mediators was also observed in human AP and recurrent AP patient samples, suggesting an involvement of inflammation in alcoholic pancreatitis [117].

There are other susceptibility factors that have been identified in experimental models and shown to be associated with alcoholic pancreatitis. Pancreatic duct obstruction, which causes minimal pancreatic damage independently, induced pancreatitis in a rat model when combined with alcohol feeding [118] and worsened the canine model of alcoholic CP [119]. Genetic mutations, as exemplified by a pathogenic human p.N256K *CPA1* (Carboxypeptidase A1) mutant when expressed in mice, caused protein misfolding, ER stress and progressive CP, which was aggravated by alcohol exposure [120]. A severe pancreatitis phenotype manifested in knock-out mice for nuclear factor erythroid 2 like 2 (NRF2), a regulator of cellular antioxidant response and ethanol metabolism, was worsened by acute binge alcohol exposure, suggesting the involvement of oxidative stress or ethanol metabolites in alcoholic pancreatitis [121].

In addition to animal models, many in vitro models have been proposed to address the mechanisms underlying the pathology of alcoholic pancreatitis. The exocrine compartment of the pancreas is mainly composed of acinar and ductal cells. The pancreatic acinar cells are the functional unit of the exocrine pancreas, constituting about 80% of the pancreas. Their function is to synthesize, store and secrete digestive enzymes. Acinar cells are believed by many to be the initiation site of pancreatic injury, as molecular and cellular events linked to acinar cell dysfunction have been shown to occur early in pancreatitis [122,123,124,125]. Similar to animal models, pancreatic acinar cells, when treated by alcohol alone, appeared to display minimal damages. Chronic alcohol exposure at a clinically relevant concentration (50 mM equivalent to 230 mg/dL, 96 h) reduced the cellular uptake of thiamine pyrophosphate (TPP) in rat primary acini, rat pancreatic AR42J acinar cells [126] and mouse pancreatic 266-6 acinar cells [127], indicative of alcohol’s damaging effects on pancreatic thiamine-dependent functions [128,129,130]. Alcohol exposure at concentrations from 200 to 800 mg/dL for 6 h caused mild apoptosis of AR42J cells and minimal effect on the activity of lipase or amylase [131]. Lugea et al. (2017) showed that alcohol treatment (50 mM equivalent to 230 mg/dL) for 4 days decreased the viability of AR42J cells only in combination with cigarette smoke extracts but not independently [132]. In CCK-8-stimulated primary mouse pancreatic acini, alcohol treatment altered Ca^2+^ homeostasis [133], increased reactive oxygen species (ROS) production [134] and reduced CCK-8-evoked amylase secretion [135]. In rat pancreatic acini, alcohol treatment exacerbated the pathological intra-acinar protease activation induced by muscarinic agonist carbachol [136].

Pancreatic ductal cells, which are responsible for transporting the acini-produced digestive enzymes into the duodenum and secreting bicarbonate-rich fluid to neutralize stomach acid, have also been proposed to be involved in the pathology of pancreatitis [137,138,139]. Alteration of ductal cell function may cause insufficient transportation or precipitation of digestive enzymes in the ducal lumen, potentially leading to obstruction and damage. Sarles et al. (1965) showed that the formation of mucoprotein plugs in the pancreatic ducts was an early lesion in the pathology of alcohol-induced chronic calcifying pancreatitis [140]. Mutations in the cystic fibrosis transmembrane conductance regulator (CFTR), an ion channel protein highly expressed in pancreatic duct cells, were found to be associated with CP [141]. Maleth et al. (2015) showed ethanol exposure reduced the expression of CFTR and disrupted the folding of CFTR at the endoplasmic reticulum (ER) in a number of human pancreatic cell lines and the pancreatic tissues of mice and guinea pigs [142]. In addition, CFTR knockout mice developed more severe pancreatitis when given ethanol than *WT* control mice [142].

## 4. Endoplasmic Reticulum (ER) Stress and Unfolded Protein Response (UPR) in Alcohol-Related Pancreatitis

The endoplasmic reticulum (ER) is an intracellular compartment that plays a major role in protein folding and processing, as well as calcium storage and release. It also serves as the first step of the secretory pathway followed by the Golgi apparatus [143,144]. Cellular stress factors, such as deficiencies in protein processing, and disturbances in calcium level or the redox state, result in the accumulation of unfolded/misfolded proteins within the ER. This is collectively known as ER stress, which triggers an adaptive response known as unfolded protein response (UPR). UPR can either resolve the ER stress when the stress is reversible or cause cell death when the stress is irreversible. The pancreatic acinar cells are particularly vulnerable to ER stress because of their primary function to synthesize and secrete digestive enzymes for food digestion, largely depending on ER functionality. ER stress and UPR signaling have been shown to be activated in a variety of experimental models of pancreatitis, including arginine-induced AP [145], caerulein- and taurocholate-induced AP [146], and CP induced by repeated episodes of caerulein [147]. The occurrence of ER stress and the activation of UPR signaling during the initiation of pancreatitis suggest that ER stress plays an important role in the development of pancreatitis. The involvement of ER stress in pancreatitis is also shown in human studies as an autosomal dominant mutation (p. R116C) in human cationic trypsinogen gene, associated with hereditary pancreatitis, which induces the accumulation of misfolded trypsinogen, ER stress and UPR signaling [148,149,150,151]. Although alcohol exposure only caused minimal pancreatic injury in animals with intact UPR functions [132,152], loss of function of a UPR regulator X-box binding protein 1 (XBP1) resulted in altered ER structure, acinar cell damage and pancreatitis-like features in alcohol-exposed animals, demonstrating a critical protective role of UPR in alcoholic pancreatitis [74,153].

**Figure 1:**
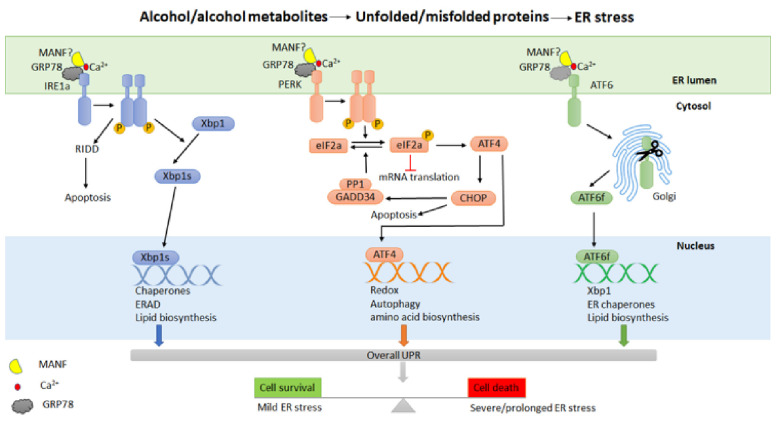
Alcohol exposure and ER stress. Alcohol and its metabolites may cause ER stress and induce a cellular adaptive response known as the unfolded protein response (UPR) in the pancreas. UPR is controlled by three transmembrane sensor proteins: inositol-requiring enzyme 1α (IRE1α), protein kinase RNA-like ER kinase (PERK) and activating transcription factor 6 (ATF6). Under non-stressed conditions, these sensor proteins bind to GPR78 and possibly MANF in a calcium-dependent manner. Alcohol exposure results in the accumulation of unfolded or misfolded proteins in the ER, in turn causing the release of GRP78/MANF to activate UPR. The activation of UPR regulates transcriptional and translational programs by restoring protein folding, promoting protein degradation, or inducing cell death.

UPR signaling is the major cellular response induced by ER stress, consisting of three distinct but also interconnected intracellular signal transduction pathways (Figure 1). These pathways are initiated by three ER-resident transmembrane sensor proteins: inositol-requiring kinase 1 (IRE1 both α and β isoforms), protein kinase-like ER kinase (PERK) and activating transcription factor 6 (ATF6 both α and β isoforms) [154,155,156]. These transmembrane sensor proteins have an ER luminal sensor domain and a cytosolic effector domain, thereby transmitting the protein folding status inside the ER to other cellular compartments via intracellular signaling pathways. In non-stressed cells, all the sensor proteins remain inactive by binding to an ER chaperone 78 kDa glucose-regulated protein (GRP78) through their N-terminus [157,158]. Under the conditions of ER stress, GRP78 dissociates from these sensor proteins, initiating their activation [157,158]. The activated UPR signaling pathways attempt to stop improper translation, facilitate protein folding and maintain ER homeostasis; however, if the ER stress is not resolved, UPR triggers cell death [159,160,161].

IRE1 is the most evolutionarily conserved ER stress sensor protein with dual protein kinase and RNase activities [162,163,164]. At the onset of ER stress, the dissociation of GRP78 activates IRE1, which involves the dimerization and trans-autophosphorylation of IRE1 kinase domains, followed by the activation of the RNase domain in the cytosol. Activated IRE1 regulates the splicing of transcription factor X box-binding protein 1 (XBP1) to generate a more stable and active form known as XBP1s [165]. XBP1s translocates to the nucleus and mediates the expression of a group of target genes in protein folding, ER-associated degradation (ERAD), and phospholipid synthesis, acting as an adaptive response that promotes the folding capacity of ER to alleviate ER stress [155,166,167]. In addition, activated IRE1 also regulates a subset of RNAs leading to cell death through a process known as IRE1-dependent RNA decay (RIDD) [168,169,170]. Both IRE1 and XBP1 are essential in secretory cells, including pancreatic acinar cells [171,172]. IRE1α conditional knock-out mice have lower pancreas mass and abnormally structured pancreatic acinar cells, but showed no difference in the level of amylase expression and secretion [172]. Conditional disruption of Xbp1 caused the decreased production of digestive enzymes and zymogen granules, altered ER structure, and extensive apoptosis in mouse pancreatic acinar cells [171,173]. In a mouse model for alcoholic pancreatitis, alcohol exposure activated IRE1/Xbp1-mediated UPR and only caused minimal pancreas damage in *WT* mice. *Xbp1^+/−^* mice displayed significant acini necrosis, inflammation, and reduction in zymogen granules and amylase levels, all indicative of the protective role of XBP1 against alcohol-induced damage in the exocrine pancreas [74].

PERK is an ER-resident kinase composed of cytosolic and kinase domains [174,175]. Similar to IRE1, the activation of PERK also involves dimerization and trans-autophosphorylation. Activated PERK phosphorylates the α-subunit of the translation initiation factor eIF2 (eukaryotic translation initiation factor-2) to reduce global protein synthesis [174,175,176]. This reduces the amount of protein entering the ER and alleviates ER stress. The phosphorylation of eIF2α by PERK also results in the selective translation of activating transcription factor 4 (ATF4), which regulates the expression of genes involved in protein folding, amino acid metabolism, and autophagy [177,178]. ATF4 also modulates the expression of proapoptotic molecules, including the transcription factor C/EBP homologous protein (CHOP) and growth arrest and DNA damage-inducible protein (GADD34) [179,180,181]. GADD34 plays a role in a feedback loop to dephosphorylate eIF2α by interacting with protein phosphatase 1 (PP1), reversing translational inhibition and inducing cell death [160,182]. PERK is highly expressed in a number of tissues, including the exocrine and endocrine pancreas [183]. PERK knock-out (*Perk^−/−^*) mice displayed a reduced expression of major digestive enzymes, abnormal ER morphology, and apoptosis of acinar cells with an increased number of stellate cells [183,184]. The loss of acinar cells and the proliferative response of stellate cells in *Perk^−/−^* mice are also often observed in patients with chronic alcoholic pancreatitis [185]. In addition, the pancreatic acinar cell-specific *Perk* knock-out mice exhibit AP-like features such as cell death and the inflammatory response [186].

ATF6 is an ER-localized membrane-bound transcription factor. Under ER stress, ATF6 is translocated to the Golgi and cleaved proteolytically to release the transcriptionally active N-terminal domain, which enters the nucleus and activates the transcription of several UPR-related genes, including GRP78, Xbp1 and CHOP [165,187,188]. ATF6 has been shown to play an essential role in modulating ER function, particularly in chronic stress [189,190]. High expression levels of ATF6, CHOP and Xbp1 have been observed in human CP pancreatic tissues, along with histological and cellular characteristics of CP, suggesting that ATF6/Xbp1/CHOP signaling may be involved in the development of CP [191]. In a CP model induced by caerulein injection in PRSS1 transgenic mice, ATF6 was shown to regulate the apoptosis of pancreatic acinar cells and the progression of CP [191].

The timing and intensity of the activation of the three UPR signaling pathways are different in response to a particular ER stressor [161,192,193]. Alcohol exposure can cause ER stress and induce UPR in the pancreas of animals and cultured pancreatic cells (Figure 1). Depending on the experimental models and the paradigm of alcohol exposure, the three pathways of UPR are differentially impacted. For example, acute alcohol exposure increased UPR components, including GRP78, p-IRE1α, XBP1, and CHOP, in human pancreatic acinar cells (hPACs) in a concentration-dependent manner [194]. Prolonged exposure to alcohol increased GRP78 and CHOP expression in AR42J cells [194]. In AR42J cells and mouse primary acini, the co-treatment with cigarette smoke extract and alcohol induced cell death, which was accompanied by PERK activation and increased expression of CHOP [132]. In animal models, it appears that a single episode of alcohol exposure is not sufficient enough to induce pancreatitis. Therefore, repeated exposure by binge drinking or combined binge and chronic alcohol exposure have been used and shown to cause pancreatitis. For example, repeated alcohol binge exposure (25% ethanol *w*/*v*, 5 g/kg/day for 10 days by oral gavage) resulted in pancreatitis-like features in male C57BL6 mice, including inflammation, increased UPR markers (ATF6, GRP78, p-PERK, p-eIF2α, and CHOP), elevated expression of amylase, and apoptosis [75]. A paradigm of chronic (5% ethanol diet for 2 weeks) plus binge alcohol exposure (5 g/kg, 25% ethanol *w*/*v* for 3 days) induced the expression of UPR markers (p-eIF2α, XBP-1, CHOP, ATF-6, and PERK), amylase secretion, pancreatic inflammation, and apoptotic cell death in the mouse pancreas [76].

## 5. Potential Treatment of Alcoholic Pancreatitis by Targeting ER Stress and UPR

**Figure 2:**
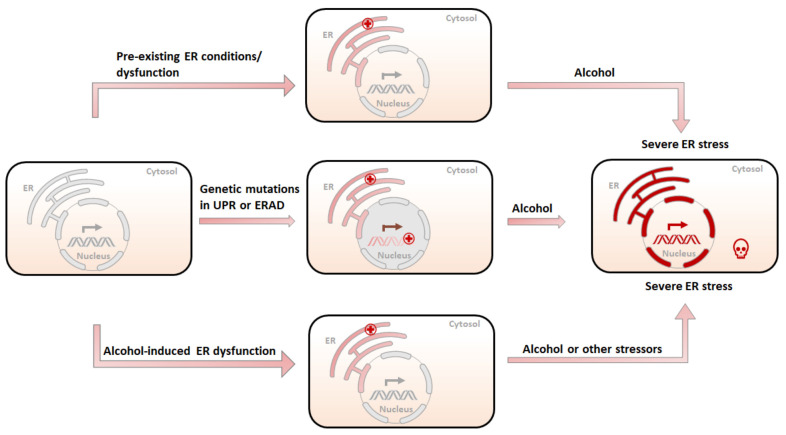
Possible etiology of alcohol-related pancreatitis. (1) A pre-existing ER condition resulting from stressors other than alcohol (tobacco, high-fat diet, etc.) is further exacerbated by alcohol exposure, causing irreversible damage to the ER and subsequent cell death. (2) Genetic mutations in UPR or ERAD compromise the ability of the ER to deal with unfolded/misfolded proteins, and therefore sensitize the ER to alcohol-induced damages, leading to severe ER stress and pancreatic damages. (3) Pre-exposure to alcohol compromises the ability of the ER to maintain homeostasis and makes the ER susceptible to subsequent alcohol exposure or other ER stressors, resulting in severe pancreatic damages.

Based on the aforementioned evidence and our own findings, we hypothesize that ER stress plays an important role in the etiology of alcoholic pancreatitis (Figure 2). Although alcohol exposure alone may not directly result in pancreatitis, it works together with other pathological conditions, such as genetic alterations and cellular stressors, to initiate the pathogenesis of pancreatitis. Alcohol may promote pancreatitis through the following mechanisms: (1) Since alcohol exposure causes ER stress in the pancreas, a pre-existing imbalance of ER homeostasis or ER dysfunction may exacerbate alcohol-induced ER stress. This is beyond UPR’s ability to restore and ultimately results in severe pancreatic damages and pancreatitis. (2) The genetic mutations or protein alterations in key components of UPR or ER-associated degradation (ERAD) pathways may already impair pancreatic cells’ ability to alleviate ER stress. Upon alcohol exposure, sustained and severe ER stress results in cell death, inflammation, and other pancreatic damages. (3) Additionally, alcohol exposure, especially chronic and heavy alcohol consumption, may disrupt ER homeostasis or impair UPR or ERAD systems, sensitizing pancreatic cells to other genetic or environmental stressors. As a result, alcohol abusers are more susceptible to etiological initiators of pancreatitis.

**Figure 3:**
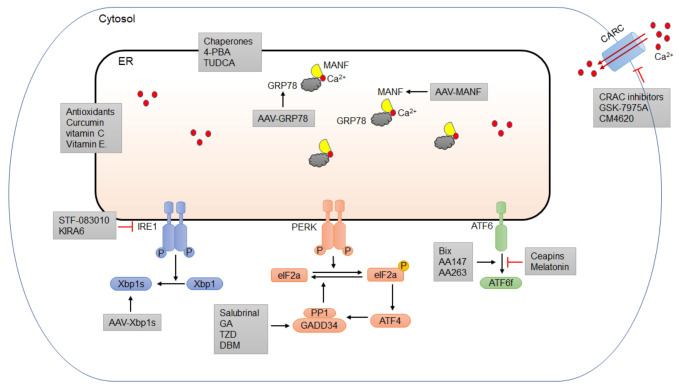
Potential pharmacological intervention for alcoholic pancreatitis targeting ER homeostasis. It is of great potential to identify specific molecules or strategies targeting ER stress and different UPR components. One of the most direct pharmacological approaches to alleviate ER stress is to use chemical chaperones, such as 4-PBA and TUDCA, to facilitate protein folding and alleviate ER stress. Another effective approach is to use specific small-molecule inhibitors or activators to modulate different UPR components. Among the three arms of UPR, PERK/eIF2α is the most important in controlling the protein translation and the transition to apoptotic cell death and has therefore drawn greater attention. A number of small molecules targeting this pathway have been shown to have protective effects against ER-stress-induced damage. Recently, several FDA-approved drugs that can affect some UPR components exhibit potential benefits to alleviate ER stress and reduce pancreatic damages. One of the potential mechanisms for alcohol-induced ER stress is the perturbation of ER calcium homeostasis. Small molecules targeting calcium channels have therapeutic potential for ER-stress-induced pancreatic damage. Antioxidants, such as vitamin C and vitamin E have been shown to alleviate ER stress and may be useful to treat alcoholic pancreatitis. Gene therapy using recombinant viruses, such as adeno-associated viruses (AAVs) is becoming an attractive strategy to deliver active UPR components to specific tissues to mitigate ER stress. AAV delivery of several key UPR proteins, such as GRP78 and MANF, demonstrates promising benefits to treat ER-stress-related tissue damage.

Since ER stress plays an important role in the pathogenesis of alcoholic pancreatitis, pharmacological modulations that target ER stress may be an effective strategy for therapy (Figure 3). Small molecules that can regulate ER homeostasis and the UPR/ERAD system have drawn great attention for this purpose (Table 1). In addition, repurposing existing drugs in a new pharmacology class is the safest and cheapest option for disease intervention. Although there are currently no FDA-approved drugs to treat alcoholic pancreatitis, a number of FDA-licensed drugs that exert therapeutic effects through controlling ER homeostasis and mitigating ER stress can be repurposed and tested for the disease [195,196,197].

### 5.1. Small Molecules

One of the most direct pharmacological approaches to alleviate ER stress is to use small molecules that function as chemical chaperones to facilitate protein folding [221]. There are several chemical chaperones, including FDA-licensed drugs such as sodium phenylbutyrate (4-PBA) and ursodeoxycholic acid (UDCA), that can be readily repurposed for the treatment of alcoholic pancreatitis (Figure 3). 4-PBA has been approved by the FDA for the treatment of patients with urea cycle disorders by acting as an ammonia scavenger [222,223]. 4-PBA can also act as an ER stress inhibitor and has been suggested to modulate the restoration of ER homeostasis in many pathological conditions [8,224,225,226]. Hong et al. (2018) showed that 4-PBA attenuated tissue injury by a reduction in the expression of ER stress markers, inflammatory response, and cell death in sodium taurocholate (ST)-induced AP in rats [198]. In addition, the trypsin activation, UPR signaling, and apoptosis of rat pancreatic acini induced by the supraphysiological cholecystokinin were suppressed by 4-PBA [199]. UDCA, also known as ursodiol, is a bile acid that has been approved by the FDA as a therapy for gallstone and liver diseases [227,228,229]. UDCA appears to have beneficial effects in treating idiopathic pancreatitis [200,201]. However, due to its poor absorption, people have recently shifted their attention to tauroursodeoxycholic acid (TUDCA), a more readily absorbed form that also has the same cytoprotective properties as UDCA. TUDCA is an ER chaperone that has been shown to attenuate ER stress and reduce intracellular trypsin activation, edema formation, and the inflammatory reaction of pancreatic tissue in a caerulein-induced AP rat model [202]. Pretreatment of TUDCA suppressed ER stress responses and alleviated ER-stress-associated apoptosis in cholecystokinin (CCK-8)-stimulated rat pancreatic acini [203].

Another approach to relieve ER stress is to manipulate the UPR pathways by using small-molecule inhibitors or repurposed FDA-licensed drugs (Figure 3). Among the three arms of UPR, PERK/eIF2α is the most important in controlling protein translation and the transition to apoptotic cell death [230,231]. Chemicals that can reduce the protein translation by modulating the PERK/eIF2α pathway are of therapeutic potential. Salubrinal is a selective inhibitor of eIF2α phosphatases that was initially identified in a screening for small molecules that protect the rat pheochromocytoma cell line PC12 from ER-stress-induced apoptosis [232]. A recent study showed that salubrinal ameliorated pancreatic injury by inhibiting the dephosphorylation of eIF2α in caerulein/LPS-induced-AP in mice [204]. However, increased eIF2α phosphorylation by salubrinal was proapoptotic in pancreatic beta cells and exacerbated the toxicity of ER stressors, such as the free fatty acids oleate and palmitate, making salubrinal an unfavorable drug candidate to treat pancreatic disorders such as alcoholic pancreatitis [233]. There are several FDA-approved drugs, including guanabenz acetate (GA), trazodone (TZD), and dibenzoylmethane (DBM), that have been shown to target different components of the PERK/eIF2α pathway and mitigate ER stress. GA is an FDA-approved anti-hypertensive drug. Trazodone is a licensed anti-depressant. DBM is a curcumin analogue that has anti-cancer properties [234]. These drugs have outstanding pharmacokinetics and are considered safe. GA has been shown to attenuate ER stress and play a beneficial role in several models of neurological diseases, including amyotrophic lateral sclerosis (ALS), oculopharyngeal muscular dystrophy (OPMD), hereditary spastic paraplegias (HSPs), and spinal cord injury (SCI) [205,206,207,208]. However, it has also been reported that GA sensitizes pancreatic β cells to fatty-acid-induced ER stress and apoptosis through PERK/eIF2α signaling [235]. TZD and DBM have been shown to provide neuroprotection and cognitive improvement by reducing protein accumulation in models of prion disease and frontotemporal dementia, with no overall toxicity [195]. In a small-molecule screening for the treatment of diabetes, TZD was identified as a stimulator for the proliferation of pancreatic β cells [209]. Despite its short-term benefit in alcohol withdrawal syndrome [236,237], TZD may increase alcohol consumption and worsen the drinking outcomes when stopped [238]. Therefore, the effects of these drugs in alcoholic pancreatitis need to be evaluated in preclinical models first.

The IRE1α/XBP1 signaling pathway is another UPR arm that has been implicated in experimental models for alcohol-induced pancreatitis [74,239]. There are two classes of small-molecule inhibitors for IRE1α that have been developed to modulate IRE1α/XBP1 signaling in ER-stress-mediated diseases [240]. The first class binds to the RNase domain of IRE1a and inhibits its RNase activity. These inhibitors, including toyocamycin, 3-ethoxy-5,6-dibromosalicylaldehyde, STF-083010, and 2-hydroxy-1-naphthaldehyde, have been shown to induce apoptosis in a number of pancreatic tumor cell lines [241]. Of note, STF-083010 has been shown to protect mouse pancreatic 266-6 acinar cells from alcohol-induced cytotoxicity in vitro [210] (Figure 3). Another inhibitor belonging to the first group, MKC-3946, was shown to cause cell death in rat pancreatic AR42J acinar cells, primary mouse, and human acinar cells in vitro [132]. The second class of IRE1α inhibitors targets its kinase domain to exert allosteric control of IRE1α RNase activity. One of the IRE1α kinase inhibitors, kinase-inhibiting RNase-attenuator 6 (KIRA6), was recently developed and shown to promote the viability and function of the pancreatic beta cells in ER-stress-induced diabetic mice [211] (Figure 3). Given the opposite effects that IRE1 inhibitors exert on cellular survival and function in different disease models, one should take precautions when repurposing them for alcoholic pancreatitis and examine their effects in experimental models on a case-by-case basis.

The modulators of ATF6 are scarce due to the unavailability of the crystal structure of the ATF6 protein, presenting challenges in the identification of druggable binding sites [242]. Using a cell-based assay, Gallagher et al. (2016) identified ceapins as a class of ATF6-specific inhibitors by preventing the translocation of ATF6 from the ER to the Golgi upon ER stress [243]. However, the effect of ceapins on the viability or function of pancreatic acinar cells has not been tested in pancreatic inflammatory contexts. Melatonin is another ATF6-selective inhibitor. In a rat model for intracerebral hemorrhage, melatonin has been shown to exert neuroprotective effects via the suppression of the ATF6 pathway [244]. Melatonin was also shown to attenuate inflammation in LPS-induced AP in AR42J cells and in taurocholate-induced AP in rats [212,213]. Interestingly, pharmacologic activation of ATF6 has also been shown to be protective against many diseases, including ischemic heart disease, diabetes, and neurodegenerative disorders [216,245,246,247,248]. Through reporter-based assays, Bix, compound 147 (AA 147) and 263 (AA 263) have been identified and both specifically activate the ATF6 arm of the UPR [214,249,250]. Bix has been shown to exert beneficial effects in experimental models for multiple disease conditions, such as stroke and kidney injury [214,215]. In a mouse model of ischemic heart disease, AA 147 was shown to exert a protective effect in multiple tissues, including heart, brain, kidney and liver [216]. These selective ATF6-activating compounds are ready to be tested in experimental models for alcoholic pancreatitis.

ER stress can also result from perturbations in calcium level, as ER resident chaperones and folding enzymes have calcium-binding sites and calcium-dependent functions [251]. Alcohol and its metabolites can deplete the calcium level in the ER by activating inositol trisphosphate receptors, calcium release channels located in the ER, to induce ER stress, alongside pancreatic acinar cell death and inflammation in experimental models for alcohol-related pancreatitis [252,253]. The release of calcium from the ER would elevate the calcium level in the cytosol, in turn activating calcium release-activated calcium (CRAC) channels on the plasma membrane to promote the uptake of extracellular calcium, further increasing the concentration of intracellular calcium. The pathological elevation of cytosolic calcium and the activated CRAC can further augment cell death and inflammation in the pancreas [137,217,252,254]. Small molecules targeting calcium channels have therapeutic potential for ER-stress-related disorders such as alcoholic pancreatitis. For example, two small-molecule inhibitors of CRAC channels (Orai1), GSK-7975A and CM_128 (also known as CM4620), have been shown to inhibit the activation of ORAI1 and prevent cell death and inflammation in thapsigargin-treated human pancreatic acinar cells and mouse models of AP induced by alcohol and palmitoleic acid [217] (Figure 3). In addition to acinar cells, CM4620 has also been shown to target pancreatic stellate cells and immune cells, block calcium entry, and reduce pancreatitis features and severity in experimental AP models [218]. In fact, CM4620 has reached Phase I clinical trials for treating AP due to its adequate specificity and low toxicity [219,220].

### 5.2. Natural-Products-Derived Antioxidants

Alcohol can also cause ER stress and pancreatic acinar cell injury by altering the redox state of the ER. Many experimental models of alcohol-related pancreatitis have shown that alcohol exposure leads to oxidative stress in the ER through its oxidative metabolites/by-products or by the generation of ROS [75,76,132,255]. Curcumin is a natural antioxidant extracted from turmeric that has been shown to protect the pancreas by lowering the severity and inflammatory response in both a rat pancreatitis model induced by alcohol and a low dose of CCK [256] and non-alcoholic pancreatitis models [256,257,258]. Due to its safeness, tolerability and low toxicity, curcumin has been tested in clinical trials for numerous diseases [259], either alone or in combination with other reagents, and it has been shown to be protective against alcohol intoxication [259] and pancreatic cancer [260,261,262]. Therefore, curcumin is a promising candidate for the treatment of alcoholic pancreatitis. Other therapeutical antioxidant candidates are vitamins that have antioxidant properties, such as vitamin C and E. Both vitamins are significantly low in the dietary intakes of patients with idiopathic CP [263] or low in the blood of patients with alcoholic AP [264] or CP [265]. Supplementation of vitamin C or vitamin E has been shown to exert anti-inflammatory and other beneficial effects in both AP patients [266] and a rat model of alcoholic CP [267].

### 5.3. Gene Therapy

Gene therapy using recombinant viruses is becoming an attractive strategy to deliver active UPR components to specific tissues. This method avoids the pleiotropic effects of systemic and chronic administration of ER-stress-targeting compounds. Adeno-associated viruses (AAVs) are the current choice to deliver therapeutic genes because of their safety profile demonstrated in pilot clinical trials [268]. GRP78 is an important ER chaperone as it participates in the regulation of all three arms of UPR signaling [269]. Enhanced GRP78 expression can alleviate ER stress in experimental models for a variety of disorders [270]. For example, AAV-mediated gene transfer of GRP78 ameliorated retinal cellular injury by mitigating ER stress in mice [271], rats [272] and human retinal epithelium cells [273]. In a rat model of Parkinson’s disease, overexpression of GRP78 by recombinant AAV attenuated ER stress, promoted the survival of nigral dopamine (DA) neurons, and restored behavioral deficits [274]. Over-production of GRP78 driven by a rat insulin promoter in pancreatic beta cells provided protection against high-fat-induced ER stress and diabetes in mice [275]. In a caerulein-induced AP model, *Grp78^+/−^* mice displayed greater pathological alterations, including morphological change, cell necrosis, edema, and inflammation, when compared with wild-type mice, suggesting a protective role of GRP78 in AP [276]. Therefore, one may take GRP78 into consideration as a potential therapeutic target in alcoholic pancreatitis, while AAV-mediated delivery of GRP78 may be readily tested in experimental models.

The downstream transcription programs of the three UPR signaling pathways are mediated by transcription factors XBP1 (IRE1 pathway), ATF4 (PERK pathway) and ATF6 (ATF6 pathway), either individually or co-operatively. Gene delivery of those transcription factors may also be a potential strategy to optimize the beneficial effects of certain pathways in different diseases. Overexpression of XBP1 in the nervous system of adult animals by viral-based delivery has been shown to exert protective effects in a mouse model for Huntington’s disease (HD) [277], spinal cord injury [278] and PD [279,280]. More recently, co-expression of XBP1 and ATF6 in a fusion protein by AAV-based delivery showed a more potent effect in the neuroprotection and anti-aggregation of mis-folded proteins than XBP1 or ATF6 alone in preclinical models for PD and HD. This suggests a cooperative action of XBP1 and ATF6 in enhancing the folding capacity of the ER and promoting cell survival under disease settings [281]. Overexpression of XBP1 by AAV-mediated delivery may be a promising therapeutic strategy readily tested in alcoholic pancreatitis because XBP1 has been implicated to have a beneficial role in alcohol-induced pancreatic damages in animals [74]. However, ATF6 has been shown to play a detrimental role in a mouse model for severe AP [282] and CP [191]. Therefore, one should remain cautious when testing the effects of its overexpression in alcoholic pancreatitis. In contrast to the beneficial effects of the overexpression of ATF6 and XBP1 in neurodegenerative disorders, the AAV-mediated overexpression of ATF4 has been shown to have deleterious effects in the brain of animal models for PD and has caused behavioral deficits when compared with the control [283]. Excessive expression of ATF4 by AAV-mediated delivery resulted in cell death associated with ER stress in mouse models for progressive retinal degeneration [284]. A more recent study showed that ATF4 contributed to the pathogenesis of AP in caerulein-induced AP mouse models [285]. Therefore, the AAV-mediated delivery of ATF4 seems to be unlikely to exert therapeutic benefits for the alcohol-induced pathology in the pancreas.

Another molecular target of interest in the treatment of alcoholic pancreatitis is mesencephalic astrocyte-derived neurotrophic factor (MANF). MANF is an ER-stress-inducible secretory protein expressed in many human and mouse tissues, with a particularly high expression level in secretory tissues such as the pancreas [286,287]. MANF is activated by alcohol exposure and plays a protective role by alleviating alcohol-induced ER stress in the brain and in cultured acinar cells [210,224,288] (Figure 3). The cytoprotective role of MANF in the pancreas has been demonstrated by the increased apoptosis and reduced proliferation of pancreatic beta cells and an insulin-deficient phenotype in pancreatic MANF knockout mice [289,290]. In humans, MANF has also been shown to be essential for ER function and proper pancreatic beta cell function [291,292]. In fact, MANF has been proposed to serve as a diagnostic biomarker for children with type I diabetes, given the elevated level of MANF found in the serum of type I diabetic children [293]. In contrast to the role of MANF in the endocrine function of the pancreas, the role of MANF in the exocrine compartment of pancreas has not drawn much attention until very recently. Using an in vitro model, we showed that an siRNA knockdown of MANF exacerbated alcohol-induced damages in mouse pancreatic 266-6 acinar cells in which the addition of recombinant human MANF or overexpression of MANF by adenovirus ameliorated alcohol-induced ER stress and cellular injury [210]. While this finding may imply a beneficial role for MANF in alcoholic pancreatitis, further studies measuring the effect of gain- or loss-of-function of MANF on pancreatitis features in animal alcoholic pancreatitis are necessary. The beneficial role of MANF is supported by the findings that the delivery of the MANF gene to the brain using AAV protected neurons against ischemic injury in animal models [294,295,296,297,298]. Therefore, it is of interest to determine whether AAV delivery of the MANF gene to the pancreas can exert protective effects against alcohol-induced damage. In addition, the serum level of MANF in patients with alcoholic pancreatitis is also worth investigating to determine if MANF can be a biomarker for alcoholic pancreatitis.

## 6. Conclusions

Today, pancreatitis remains a serious medical concern worldwide, with no FDA-approved drugs or treatments available for the disease. Both AP and CP are commonly caused by excessive alcohol use along with other risk factors, including smoking, high-fat diet and genetic mutations. The prevention of the disease primarily consists of alcohol and smoking cessation and a change to a pancreas-friendly diet.

ER stress has been documented in numerous experimental models as an early event in alcohol-induced damage to pancreatic acinar cells. Approaches targeting ER stress may open a new avenue for therapeutic strategies for the disorder. For example, small molecules, FDA-approved chemicals, and gene therapy aiming at restoring ER homeostasis have shown some promising effects in treating the disease. Further study of clinical trials and investigation into each specific pathway such as UPR, ERAD and autophagy in maintaining/restoring ER homeostasis could provide insight into novel therapeutic strategies and potential biomarkers in predicting clinical outcomes.

## Figures and Tables

**Table 1:** Candidate small molecules targeting ER stress for the treatment of alcohol-related pancreatitis.

Molecule	Experimental Model/Clinical Settings	Effects	Ref.
Chemical chaperones			
Sodium phenylbutyrate (4-PBA)	CCK-stimulated rat pancreatic acini; taurocholate-induced AP rats	Rescue cell death; protect pancreas	[198,199]
UDCA	Idiopathic AP and recurrent pancreatitis patients	Remove gallstones and prevent pancreatitis relapse	[200,201]
Tauroursodeoxycholic acid (TUDCA)	CCK-8-stimulated rat pancreatic acini; caerulein-induced AP rats	Reduce cell death, trypsin activation and edema formation	[202,203]
PERK/eIF2α inhibitors			
Salubrinal	Caerulein/LPS-induced-AP mice	Reduce serum amylase level, inflammation and cell death	[204]
Guanabenz acetate (GA)	Mouse model of neurological diseases	Exert neuroprotection	[205,206,207,208]
Trazodone (TZD)	Small-molecule-screen for β-cell proliferation in transgenic zebrafish	Stimulate proliferation of pancreatic β cells	[209]
Dibenzoylmethane (DBM)	Mouse models of dementia	Improve neuroprotection and cognition; no toxicity to pancreas	[195]
IRE1α Inhibitors			
STF-083010	Alcohol-treated mouse pancreas 266-6 acinar cells	Reduce cell death	[210]
Kinase-Inhibiting RNase-Attenuator 6 (KIRA6)	Akita diabetic mice	Reduce cells death of pancreatic islets in vitro and in vivo	[211]
ATF6 inhibitors			
Melatonin	LPS-treated rat AR42J acinar cells; taurocholate-induced AP rats	Attenuate inflammation, reduce apoptosis	[212,213]
Bix	Gerbil model of forebrain ischemia; mouse model of renal I/R injury	Rescue cell death	[214,215]
Compound 147 (AA 147)	Mouse model of acute myocardial infarction	Cytoprotective effects in heart, brain, kidney and liver	[216]
Inhibitors of CRAC channels			
GSK-7975A	AP mice induced by TLCA3S, caerulein or ethanol and palmitoleic acid	Reduce serum amylase level, cell death and inflammation	[217]
CM_128/CM4620	Phase I clinical trials for AP		[218,219,220]

## Data Availability

Not applicable.

## Abbreviations

AP	acute pancreatitis
CP	chronic pancreatitis
ER	endoplasmic reticulum
UPR	unfolded protein response
EPI	exocrine pancreatic insufficiency
PC	pancreatic cancer
DM	diabetes mellitus
CLDN2	clauding 2
CTRB1	chymotrypsin B1
CTRB2	chymotrypsin B2
ALDH2	aldehyde dehydrogenase-2
ADH1B	alcohol dehydrogenase-1B
ALD	alcoholic liver disease
ALT	alanine aminotransferase
AST	aspartate aminotransferase
LPS	lipopolysaccharides
CPA1	carboxypeptidase A1
TPP	thiamine pyrophosphate
ROS	reactive oxygen species
CFTR	cystic fibrosis transmembrane conductance regulator
IRE1	inositol-requiring kinase 1
PERK	protein kinase-like ER kinase
GRP78	78 kDa glucose-regulated protein
eIF2	eukaryotic translation initiation factor-2
XBP1	X box-binding protein 1
ERAD	ER-associated degradation
RIDD	IRE1-dependent RNA decay
ATF6	activating transcription factor 6
ATF4	activating transcription factor 4
CHOP	C/EBP homologous protein
GADD34	growth arrest and DNA damage-inducible protein 34
MANF	mesencephalic astrocyte-derived neurotrophic factor
hPAC	human pancreatic acinar cells
ERAD	ER-associated degradation
4-PBA	sodium phenylbutyrate
UDCA	ursodeoxycholic acid
TUDCA	tauroursodeoxycholic acid
CCK-8	cholecystokinin-8
GA	guanabenz acetate
TZD	trazodone
DBM	dibenzoylmethane
ALS	amyotrophic lateral sclerosis
OPMD	oculopharyngeal muscular dystrophy
HSPs	hereditary spastic paraplegias
SCI	spinal cord injury
AA 147	compound 147
AA 263	compound 263
CRAC	calcium release-activated calcium channel
AAV	adeno-associated virus
DA	nigral dopamine
TLCA3S	taurolithocholic acid 3-sulfate
